# Identifying the minimal important difference in patient-reported outcome measures in the field of people with severe mental illness: a pre–post-analysis of the Illness Management and Recovery Programme

**DOI:** 10.1007/s11136-021-02779-4

**Published:** 2021-02-17

**Authors:** Titus A. A. Beentjes, Steven Teerenstra, Hester Vermeulen, Peter J. J. Goossens, Maria W. G. Nijhuis-van der Sanden, Betsie G. I. van Gaal

**Affiliations:** 1 Radboud University Medical Center, Radboud Institute for Health Sciences, IQ Healthcare, Nijmegen, The Netherlands; 2 Center for Nursing Research, Saxion University of Applied Science, Deventer/Enschede, The Netherlands; 3 Dimence Group Mental Health Care Centre, Deventer, The Netherlands; 4 Radboud University Medical Center, Radboud Institute for Health Sciences, Department for Health Evidence, Group Biostatistics, Nijmegen, The Netherlands; 5 University Centre for Nursing and Midwifery, Department of Public Health, Faculty of Medicine and Health Sciences, Ghent University, Ghent, Belgium; 6 HAN University of Applied Sciences, Faculty of Health and Social Studies, Nijmegen, The Netherlands

**Keywords:** Self-management, Recovery, Minimal important difference, Severe mental illness, Illness management and recovery

## Abstract

**Purpose:**

Complementary interventions for persons with severe mental illness (SMI) focus on both personal recovery and illness self-management. This paper aimed to identify the patient-reported outcome measures (PROMs) associated with the most relevant and meaningful change in persons with SMI who attended the Illness Management and Recovery Programme (IMR).

**Methods:**

The effect of the IMR was measured with PROMs concerning recovery, illness self-management, burden of symptoms and quality of life (QoL). From the QoL measures, an anchor was chosen based on the most statistically significant correlations with the PROMs. Then, we estimated the minimal important difference (MID) for all PROMs using an anchor-based method supported by distribution-based methods. The PROM with the highest outcome for effect score divided by MID (the effect/MID index) was considered to be a measure of the most relevant and meaningful change.

**Results:**

All PROMs showed significant pre**–**post-effects. The QoL measure ‘General Health Perception (Rand-GHP)’ was identified as the anchor. Based on the anchor method, the Mental Health Recovery Measure (MHRM) showed the highest effect/MID index, which was supported by the distribution-based methods. Because of the modifying gender covariate, we stratified the MID calculations. In most MIDs, the MHRM showed the highest effect/MID indexes.

**Conclusion:**

Taking into account the low sample size and the gender covariate, we conclude that the MHRM was capable of showing the most relevant and meaningful change as a result of the IMR in persons with SMI.

## Introduction

In recent decades, the focus of treatment for people with severe mental illness changed from decreasing the burden of symptoms towards living a meaningful life [1]. In the 1980s, the concept of recovery was introduced, defined as a deeply personal, unique process of changing one’s attitudes, values, feelings, goals, skills and/or roles [2]. In the 1990s, as a result of better general health care, life expectancy grew, illnesses became chronic, the challenge to manage chronic illnesses and their consequences increased and the term ‘self-management’ was introduced [3].

In the field of people with severe mental illness, self-management and symptom reduction represent the clinical orientation, whereas recovery is used as an orientation for personal issues [1, 2]. In this field, a dismissive attitude towards labelling mental illnesses can be heard because of the stigmatizing tendencies [1, 4]. Several interventions with a single focus on recovery have been developed to help persons with severe mental illness to choose, acquire and keep valued roles. Complementary interventions provide both illness self-management and personal recovery-orientated strategies [5]. An example of a complementary intervention is the Illness Management and Recovery (IMR) programme [6]. Internationally the IMR programme is criticized for its too dominant clinical orientation and McGuire et al. [7] recommend exploring the effects of the IMR programme on recovery and severity of symptom outcomes. In different trials, the IMR programme showed effects on patient-reported outcome measures (PROMs) in domains of recovery [8–10], symptom reduction [8, 11] and illness self-management [12–14].

A PROM is defined as any report coming directly from patients about how they function or feel in relation to a health condition and its therapy [15]. PROMs are considered to be able to measure clinically relevant pre**–**post-effects from a patient perspective. To assess if a change in pre**–**post-measures is relevant and meaningful, the concept of minimal important difference (MID) is introduced [16–18]. Guyatt et al. [19] defined the MID as the smallest difference in outcome in the domain of interest that patients perceive as important, either beneficial or harmful. Knowing that an intervention can enhance an important difference in a desired outcome domain may help patients, caregivers and professionals when considering shared decision-making processes. King [20] states that MIDs can convince clinicians to change their treatment practices and convince policy-makers to change their treatment guidelines. The concept of MID has become a standard approach in determining the clinical relevance of changes in PROMs. No scientific literature on MID for PROMs concerning people with severe mental illness are available. In this study, we want to contribute to knowledge about MID in the field of severe mental illness.

Considering the discourse of a clinical versus personal recovery orientation in the field of people with severe mental illness, this paper aimed to identify the PROM that captures the most relevant and meaningful change as a result of the IMR programme in persons with severe mental illness. If we are aware of this we are able to measure more uniformly in clinical practice and in research.

## Materials and methods

### Trial design, settings and study population

We performed pre- and post-tests to measure the effect of attending the IMR programme on different PROMs. To examine which PROM captured the most relevant and meaningful change, we used the concept of the MID [17, 20–22]. Estimating MIDs of the PROMs was based primarily on an anchor-based method but supported by distribution-based methods. We used an additional index to assess whether the effect of the IMR programme in a given PROM was large or small seen from a patient perspective, hence in terms of the MID. In this way, we arrived at the effect/MID as an index. An effect/MID of ≥ 1 indicates that the effect is at least the MID: the higher the index the more patients have a change score above the MID. Typically, within-patient changes are normally distributed around the mean change at group level. By comparing the index across different outcome measures, we could identify on which PROM the participants improved the most from the IMR programme. We considered that the PROM with the highest effect/MID index represents the most relevant and meaningful change as a result of the IMR programme. The study population consisted of participants from the e-IMR trial [23, 24]. Eligible participants met the following criteria: above 18 years of age, capable of giving informed consent and meeting the Dutch severe mental illness criteria according to Delespaul [25]: being diagnosed with a psychiatric disorder that causes, and is due to, serious impairment in social and/or occupational functioning that lasts longer than at least a couple of years and necessitates coordinated multidisciplinary care. The IMR programme was delivered groupwise in Dutch mental health institutions and lasted about 1 year for all the groups. Further information on the e-IMR trial is published elsewhere [23, 24].

### Data collection and outcome measures

In this study, we used the pre**–**post-data that were gathered in the e-IMR trial between January 2015 and October 2016 [23]. The first author and research assistants sampled all data in structured interviews. Face-to-face interviews were held, because we estimated that too many participants would not respond to telephone or online questionnaires. In the population of people with severe mental illness, low computer experience, skills [26] and availability [24] exist. Furthermore, this population is known for their cognitive impairments [27]. We decided to use the advantages of face-to-face interviewing: spoken language that can be better understood; the possibility of responding to misunderstanding and probing for complete answers and using cards with answering options to overcome memorizing difficulties [28]. The interviewers tried to create an easy-going atmosphere using small talk between the different questionnaires. The interviews took 30–60 min and data were sampled using the following six PROMs:

1. Illness management was measured with the *Illness Management and Recovery Scales* (IMRS), consisting of 15 items. The response levels, on a 5-point scale (1–5), vary depending on the item. The total score ranges from 15 to 75 and a higher score indicates a higher level of illness management [29]. The test**–**retest coefficient (*r*_xx_) for the IMRS varies between 0.79 and 0.84 [30–33].
2. Self-management was measured with the *Patient Activation Measure* (PAM), consisting of 13 items. The response levels, on a four-point scale, vary from ‘strongly disagree’ to ‘strongly agree’, and the fifth option is ‘not applicable’. Raw scores were transformed into scores ranging between 0 and 100 and a higher score indicates a higher level of self-management [34]. Coefficient *r*_xx_ for the PAM is 0.76 [35].
3. Recovery was measured with the *Mental Health Recovery Measure* (MHRM), consisting of 30 items. The response levels, on a five-point scale, vary from ‘strongly disagree’ (0) to ‘strongly agree’ (4), with ‘neutral’ in between (2). The total score ranges from 0 to 120 and a higher score indicates a higher level of recovery [36]. Coefficient *r*_xx_ for the MHRM is 0.92 [37].
4. Burden of symptoms was measured with the *Brief Symptom Inventory* (BSI), consisting of 53 items. The response levels, on a five-point scale, vary from ‘not at all’ (0) to ‘extremely’ (4) [38]. The mean score ranges from 0 to 4 and a higher score indicates a higher level of burden and a lower level of mental health. Coefficient *r*_xx_ for the BSI is 0.90 [39].
5. Quality of life (QoL) was measured with the *Manchester Short Assessment of Quality of Life* (MANSA), rating satisfaction with your life as a whole as the first item (MANSA-1) and 11 other items focusing on social, physical and mental health domains on a 7-point scale, varying from ‘couldn’t be worse’ (1) to ‘couldn’t be better’ (7). The mean score ranges from 1 to 7 and a higher score represent a higher level of QoL [40]. Coefficient *r*_xx_ for the MANSA is 0.82 [41].
6. Quality of Life was also measured in terms of general health with the *Rand 36-Item Health Survey*, consisting of 36 items assembled into nine concepts. Raw scores in all the concepts were transformed into scores ranging between 0 and 100, in steps of 5. A higher score indicates a higher level of health [42]. In this study, we used only two concepts: General Health Perception (Rand-GHP) and Health Change (Rand-HC). Rand-GHP consists of five items that estimate the participant’s current perception of general health (bio-psycho-social): (1). how many times the participants’ health status hindered them in social activities, which was scored on a five-point scale (*all, most, some, a little, none of the time*); (2) whether they estimate that they become ill more easily than other people; (3) whether they estimate that their health status is just like other people they know; (4) whether they expect their health status to decline; and (5) whether they estimate that their health status is excellent. Items 2–5 were scored on a five-point scale (*definitely true, mostly true, don’t know, mostly false, definitely false*). Coefficient *r*_xx_ is 0.80 for Rand-GHP [43]. Rand-HC consists of one item that estimates health compared to a year ago on a five-point scale (*much better, better, the same, worse, much worse*). At the endpoint of our study, this ‘year ago’ was the start of the IMR programme. Coefficient *r*_xx_ is 0.40 for the Rand-HC [43].

During the pre-test, participant characteristics were also sampled: age, gender, psychiatric diagnoses conforming to DSM-IV criteria, psychiatric and somatic comorbidities, treatment history, cultural background, housing, socioeconomic status and highest education (see Table 1). At the endpoint, non-completers were identified as those who attended fewer than 50% of the IMR programme sessions.

**Table 1:** Participant characteristics

Variable	*n*(%)	Mean (SD)
*Pre-test participant characteristics*	60 (100)	
Female	36 (60)	
Age		45 (11.6)
*Diagnoses*		
Psychotic disorders	20 (33)	
Mood/anxiety disorders	25 (42)	
Other disorders	15 (25)	
Global Assessment of Functioning	50.5 (8.7)	
Having a somatic comorbidity	30 (50)	
Having a psychiatric comorbidity	38 (63)	
*Treatment history*		
Years ago since first treatment		16.9 (11.5)
Number of admissions		4 (3.7)
Never admitted	9 (15)	
*Housing*		
Independent living	38 (63)	
Living in supported housing facility	22 (37)	
*Netto income*		
≤ Minimal income	47 (78)	
> Minimal income	13 (22)	
*Highest graduated education*		
≤ Middle school	38 (63)	
≥ High school	22 (37)	
*Post-test participant characteristics*	45 (75)	
Female	25 (56% out of 45)	
Non-completers	14 (31% out of 45)	

*n* number, *SD* standard deviation

### *Method for investigating pre*–*post-effects*

Analyses were conducted using SPSS software, version 23 [44]. To determine the pre–post-effects of the IMR programme on all PROMs, we performed mixed-model multilevel regression analyses, taking into account the clustering of participants. Because the IMR programme was delivered via group sessions, the participants were clustered in these groups. This method automatically uses the ‘missing at random’ assumption to handle missing data. Random effects on cluster and individual participants nested within the cluster and fixed main effects for time trend were included in the model. The analyses were executed according to the intention-to-treat principle. To take into account the influence of covariates, the pre–post-effects were controlled for the participant characteristics one by one, including for non-completers. The pre–post-effects in the PROMs were used to estimate the effect/MID index.

### Methods for investigating the MID

To estimate a MID, both anchor-based and statistical distribution-based methods are recommended [17, 20–22]. The anchor-based method uses a criterion that measures a concept that health professionals are familiar with and is widely used in assessing patients’ health status [20], such as clinical endpoints, global transition questions or QoL measures [17]. As there is no such widely used criterion in mental health, we searched for a criterion in our own data that captures the richness and variation of the construct of a QoL measure [17]. We examined four QoL anchor candidates: the endpoint data on the Rand-HC global transition question and the change scores on MANSA-1, the total MANSA and Rand-GHP. Rand-HC and MANSA-1 were only used in the search for an anchor. The strength of the association between the anchor and the PROM needs to be determined, because low or no correlation can provide misleading information [17, 45]. A correlation of at least 0.30–0.35 is recommended [17], therefore, Spearman’s correlation coefficients between the anchor candidates and the PROMs were calculated. Outliers should not drive a correlation to a significant level. In SPSS, scores above 2.58 times the standard deviation (SD) are assigned as probable outliers [46]. Probable outliers were assessed on their appropriateness and impact on the correlation and a decision was made about removing or recoding to a reasonable level [47, 48]. The anchor candidate with the highest correlation with the change scores in most PROMs was considered to be the best anchor and, therefore, will be used in the MID-anchor calculations.

Estimation of the MID-anchor proceeds as follows: the scores on the anchor were used to categorize participants into five groups that reflected relevant and meaningful change (*large negative, small negative, no, small positive, large positive*); the mean of the four differences between change scores in the PROMs for two succeeding change groups is the PROM’s MID-anchor [20]. The MID-anchor was used to estimate the effect/MID-anchor index.

It is recommended that the MID be estimated primarily by anchor-based methods [17, 49] and to use distribution-based methods as supportive information [17]. Therefore, we examined two statistical distribution-based MID methods based on the effect size (ES) and standard error of measurement (SEM) of the PROM [17]. The ES estimates the effect of the intervention related to the SD, with ½ES as standard for estimating the MID [17, 20–22, 50]. In MID studies, the SDs of the baseline scores and change scores are used to estimate the ES [21, 50]. A change score is the endpoint score minus the baseline score of a participant. The SD of the change scores (SD_c_) relates to between-patient variation in change scores. Our index (effect/MID-SD_c_) is 2 × effect/SD_c_, which is also known as the standardised response mean [45, 51]. To calculate the effect/MID-SD_c_ index, we used the estimated effects from the mixed-model analyses.

The SEM is computed using the SD and the test–retest coefficient index: SD × √(1 − *r*_xx_) [20, 28, 47, 52]. To estimate the SEM of the PROMs used in our study, we used the SD and *r*_xx_ reported in psychometric studies of the PROMs in populations comparable to ours as much as possible. A change smaller than the SEM is likely to be a result of the measure’s unreliability rather than a true observed change, therefore, the PROM’s MID based on the SEM (MID-SEM) is equal to the SEM of the PROM. The MID-SEM and the estimated effects from the mixed-model analyses were used to estimate the effect/MID-SEM index.

When covariates modified the effects, we re-estimated the effect/MID indexes and stratified the participants according to the modifying covariate.

## Results

### Participant flow

Ten IMR programme groups entered the trial, totalling 91 potential participants, 60 of whom (66%) participated. Baseline characteristics of the participants are presented in Table 1: 36 participants (60%) were female and 15 (25%) were lost to follow-up. In total, 45 participants completed the post-test measurements, 25 (56%) of whom were female; 14 (23%) were non-completers, meaning that they attended less than 50% of the IMR programme sessions.

### Pre–post-effect analyses

Table 2 shows the estimated effects, which were significant for all the PROMs: illness management (IMRS), recovery (MHRM), self-management (PAM), burden of symptoms (BSI), QoL (MANSA) (*p* < 0.01) and general health perception (Rand-GHP) (*p* < 0.05). Only the gender covariate modified effects significantly for Rand-GHP (*p* < 0.01). For males, the estimated effects were significant only for the MHRM (*p* < 0.05), whereas for females, they were significant for all the PROMs (*p* < 0.01; see Table 2).

**Table 2:** Analyses of Mixed model effects, standard deviation of the change scores and analyses of Minimal Important Differences, and effect/MID indexes in total population and genders separately

Measures	Mixed model estimated effects	Mixed model estimated effects on gender	SD_c_	MID-anchor	Effect/MID- anchor index	MID-SD_c_	Effect/MID- SD_c_ index	MID-SEM	Effect/MID- SEM index
*Analyses for total population (n* = *60)*									
IMRS	3.36^**^	− 2.01	6.62	2.9	1.14	3.3	1.02^¶^	3.2	1.07^¶^
PAM	5.70^**^	2.0	12.32	5.7	1.00	6.2	.92	7.0	.82
MHRM	7.93^**^	− 2.30	11.90	6.1	1.29^¶^	6.0	1.33^¶^	5.7	1.40^¶^
BSI	− .17^**^	.28	.51	.25	.89	.29	.75	.23	.74
MANSA	.22^**^	− .27	.58	− .23	.74	.26	.66	.46	.47
Rand-GHP	4.73^*^	− 11.54^**^	17.43	15.5	.31	8.7	.54	10.2	0.47
*Analyses for males* (*n* = 24)									
IMRS	.92		5.61	3.22	.28	2.81	.33	3.2	.29
PAM	5.70		14.28	6.48	.88^¶^	7.14	.80	7.0	.82
MHRM	5.02^*^		12.11	7.44	.67	6.06	.83^¶^	5.7	.89^¶^
BSI	.03		.30	.16	− .19	.15	− .19	.23	− .13
MANSA	.01		.55	.35	.04	.28	.05	.46	.03
Rand-GHP	− 4.31		15.80	16.67	− .26	7.90	− .55	10.2	− .42
*Analyses for females* (*n* = 36)									
IMRS	5.08^**^		6.99	2.28	2.23	3.50	1.45	3.2	1.61
PAM	5.55^**^		10.80	4.79	1.16	5.40	1.03	7.0	.80
MHRM	9.93^**^		11.62	3.78	2.63^¶^	5.81	1.71^¶^	5.7	1.76^¶^
BSI	− .31^**^		.60	− .29	1.09	.30	1.03	.23	1.37
MANSA	.37^**^		.56	.15	2.37	.28	1.29	.46	.80
Rand-GHP	11.52^**^		16.33	9.44	1.22	8.16	1.41	10.2	1.14

*MID* minimal important difference, *IMRS* Illness Management and Recovery Scales, *PAM* Patient Activation Measure, *MHRM* Mental Health Recovery Measure, *MANSA* Manchester Short Assessment of Quality of Life, *BSI* Brief Symptom Inventory, *Rand-GHP* Rand General Health Perception, *n* number of participants, *SD*_*c*_ standard deviation of the change scores, *SEM* standard error of measurement

^*^Effect is significant at the *p* < .05

^**^Effect is significant at the *p* < .01

^¶^Highest effect/MID index

### Analysis of the MID

The results of the MID analyses are shown in Table 2. The change scores on all the PROMs were normally distributed. The health change measure (Rand-HC) showed no significant correlations to the other PROMs and was therefore considered to be a non-feasible anchor. Compared to the QoL measures MANSA-1 and the MANSA, the general health measure Rand-GHP showed the most frequent, highest and statistically significant (*p* < 0.01) correlations with the following measures: illness management (IMRS), self-management (PAM), recovery (MHRM), burden of symptoms (BSI), and QoL (MANSA) (see Table 3). Two outliers drove the correlation between Rand-GHP and the BSI. Examination identified the outliers as true scores and to assess their impact on correlation they were recoded into twice the *SD*_c_ (= 1.02), after which the correlation remained significant (*p* < 0.01; see Table 3). Rand-GHP was selected as the anchor.

**Table 3:** Spearman’s correlations between change scores of patient reported outcome measures

Measures	PAM	MHRM	BSI	Quality of Life measures			
				MANSA-1	MANSA	Rand-HC	Rand-GHP
IMRS	.246	.499^**^	− .428^*^	− .069	.570^**^	.183	.532^**^
PAM		.433^**^	− .242	− .003	.162	.112	.511^**^
MHRM			− .540^**^	.147	.340^*^	.208	.480^**^
BSI				− .140	− .346^*^	− .121	− .390^**^
BSI^¶^				− .138	− .350^*^	− ,120	− .384^**^
MANSA-1					.308^**^	− .059	.104
MANSA						− .023	.477^**^
Rand-HC							.204

*IMRS* Illness Management and Recovery Scales, *PAM* Patient Activation Measure, *MHRM* Mental Health Recovery Measure, *MANSA-1* first question of the Manchester Short Assessment of Quality of Life, *MANSA* Manchester Short Assessment of Quality of Life, *BSI* Brief Symptom Inventory, *Rand-HC* Rand Health Change, *Rand-GHP* Rand General Health Perception

^*^Correlation significant at the *p* < 0.05 (two-tailed)

^**^Correlation significant at the *p* < 0.01 (two-tailed)

^¶^BSI with outliers corrected to 2*SD_c_

We categorized the participants in five change groups. The change scores in the Rand-GHP vary from − 35 to + 40 in steps of 5 (see Table 4). To form the five change groups, we estimated that each group consists of participants that have three consecutive scores in steps of 5, so the score difference between succeeding change groups was 15, which is comparable to the SD_c_ of 17.4. In the ‘no change’ group, we categorized participants with change scores around zero (− 5, 0, 5). We subsequently defined the other groups and the remaining score + 40 was assigned to the ‘large positive change’ group. This resulting five change groups are: large negative (*n* = 3), small negative (*n* = 7), no (*n* = 18), small positive (*n* = 10), and large positive change (*n* = 7) (see Table 4).

**Table 4:** Participants’ change scores on Rand-GHP categorized in five subgroups of the total population, and in four gender subgroups

Change groups	Change score	*n* *n* per group		*n* males *n* per group		*n* females *n* per group	
Large negative change	− 35	1		1		0	
	− 30	0		0		0	
	− 25	2	3	2	3	0	
Small negative change	− 20	2		1		1	
	− 15	1		0		1	
	− 10	4	7	3	4	1	3
No change	− 5	6		4		2	
	0	7		3		4	
	5	5	18	3	10	2	8
Small positive change	10	4		0		4	
	15	5		2	3	3	
	20	1	10	0		1	8
Large positive change	25	1		0		1	
	30	1		0		1	
	35	4		1		3	
	40	1	7	0		1	6
Total		45	45	20	20	25	25

*Rand-GHP* Rand General Health Perception, *n* number

The effect/MID-anchor index was highest in the recovery measure (MHRM), with a value of 1.29.

Regarding the supportive MIDs, the MID-SD_c_ was calculated for the PROMs (see Table 2) and the effect/MID-SD_c_ index was highest in the MHRM with a value of 1.33. The MID-SEM values for the PROMs were equal to the SEM, which we calculated for the PROMs with data from referent studies (see Table 5). The effect/MID-SEM index was also highest in the MHRM with a value of 1.40.

**Table 5:** Analyses of the standard error of measurement with data from the referent studies (SEM = SD × √(1 − *r*_xx_))

Measures [references]	r_xx_	SD	SEM
IMRS [31]	.80	6.99	3.15
PAM [35]	.76	14.21	6.96
MHRM [37]	.92	20.00	5.66
BSI [39]	.90	.72	.23
MANSA [41]	.82	1.08	.46
Rand-GHP [43]	.80	22.7	10.15

*SEM* Standard error of measurement, *SD* standard deviation, *r*_*xx*_ test–retest coefficient, *IMRS* Illness Management and Recovery Scales, *PAM* Patient Activation Measure, *MHRM* Mental Health Recovery Measure, *BSI* Brief Symptom Inventory, *MANSA* Manchester Short Assessment of Quality of Life, *Rand-GHP* Rand General Health Perception

Owing to the modifying effect of the gender covariate, we also stratified the MID calculations (see Table 2). For the stratified calculations of the MID-anchor, we reorganized the change groups into four groups based on the 15-point difference, because the male/female proportions in the different change groups were skewed (see Table 4). Males were overrepresented in the negative change groups and females were overrepresented in the positive change groups. Only one male was present in the large positive change group and females were absent in the large negative change group. For the groups with male participants we merged the two positive change groups, resulting in large negative (*n* = 3), small negative (*n* = 4), no (*n* = 10) and positive (*n* = 3) change groups; for the groups with female participants, we omitted the large negative change group, resulting in small negative (*n* = 3), no (*n* = 8), small positive (*n* = 8) and large positive (*n* = 7) change groups. The mean differences between the four change groups estimated the MID-anchors for males and females separately. The effect/MID-anchor index was highest for males (0,88) in the self-management measure (PAM) and for females (2,63) in the recovery measure (MHRM).

For the supportive MIDs, the MID-SD_c_ was stratified for males and females. The effect/MID-SD_c_ indexes for both males (0.83) and females (1.71) were highest in the MHRM. The MID-SEM for males and females separately was not calculated, because the referent studies did not provide data for males and females. The effect/MID-SEM indexes for both males (0.89) and females (1.76) were highest in the MHRM.

## Discussion

Considering the discourse of a clinical versus personal recovery orientation in the field of people with severe mental illness, this paper aimed to identify the PROM that captures the most relevant and meaningful change as a result of the IMR programme in persons with severe mental illness. In the whole study population, the recovery measure (MHRM) showed the highest effect/MID index in all the MIDs. Also, in the subgroups stratified by gender, the MHRM had the highest effect/MID index in nearly all the MIDs except for the effect/MID-anchor index for men, which was highest in the self-management measure (PAM). With certain prudence, we conclude that the MHRM captures the most relevant and meaningful change for persons with severe mental illness.

Pre–post-scores improved statistically significantly on all the PROMs. The improvements in self-management (PAM) and illness management (IMRS), are bigger than the decrease of burden of symptoms (BSI). The improvements in illness- and self-management might have enhanced their perceived recovery more than symptom reduction. This matches with Slade’s statement that self-management is related to recovery because it can be a vital resource for supporting recovery [1]. Our findings showed that the IMR programme is capable of facilitating recovery using both illness self-management and personal recovery-orientated strategies, which is also claimed in previous research [7, 53, 54]. In another earlier study on this population, we saw that women scored better than men, as if women could benefit more from the IMR programme than men [23]. However, before concluding that the IMR programme should be preserved for females, we suggest re-investigating the possible difference in effect between men and women in a larger trial sample.

The overall results on effect/MID index indicate that participating in the IMR programme brought about an important change in the participants. However, on none of the PROMs did the male participants score an effect/MID index of > 1. Considering the concept of the MID, Revicki et al. [45] state that the ½SD magnitude of change is certainly clinically significant but may not be the smallest non-ignorable difference: ½SD in an outcome measure might be too large to be considered minimally important [45]. Revicki et al.’s statement might also apply for ½SD_c_, because in our study, the SD_c_ and the SD on baseline scores differed only slightly. Although the mean change in the male participants in our study was < 1MID, we saw that they improved significantly on the recovery measure (MHRM).

On comparing the three calculated MIDs in the PROMs, we conclude that they do not differ by very much. Similarity of the distribution-based MIDs would be expected when the reliability index *r*_xx_ is 0.75 in a SEM calculation, because then both the MID-SD_c_ and the MID-SEM are equal [52]. The *r*_xx_ in the main PROMs in our study ranged between 0.76 and 0.92. When *r*_xx_ is higher than 0.75, the MID-SEM is expected to be lower than the MID-ES. In our study, this is the case in the MHRM and the BSI but not in the QoL measure MANSA due to the difference between the SD in the reference study [41] and the SD_c_ in our study. Nevertheless, in our study, the results in the three MIDs are reasonably consistent and therefore we can conclude that the MID-SD_c_ and MID-SEM support the findings on the MID-anchor.

The anchor-based method is our preferred method, as also recommended by Revecki et al. and Johnstone et al. [17, 49]. Jayadevappa et al. [15] mention there is no agreement regarding appropriate anchors. The health change (Rand-HC) ‘global transition question’ anchor-based method appeared to be non-feasible, which is in line with other studies that declared inaccuracy related to response shifts and recall bias [20, 55, 56]. Recall bias might also be responsible for Rand-HC’s low test–retest coefficient (*r*_xx_ = 0.40) found in the study of Van der Zee et al. [43]. Nevertheless, this global transition question is still recommended for estimating the MID.

In our study, we found the general health perception measure (Rand-GHP) to be the best anchor. Although this choice was data driven, we also considered that Rand-GHP captures the richness and variation of a construct of QoL. The five Rand-GHP questions contain important issues in estimating one’s health status: global estimations of whether their health status hinders them in social activities, whether their health status is excellent, whether they expect deterioration and two questions on whether one’s health differs compared to the persons they know. There is considerable evidence that evaluating oneself favourably in comparison with others is associated with having fewer health problems [57]. Social comparison also is an important behavioural change technique [58]. Because of the groupwise deliverance of the IMR programme in our study, participants became acquainted with peers. Comparing oneself to peers might be more realistic than a comparison to healthy persons. Perceiving one’s health status as deteriorating is associated with a higher need for support with self-management tasks [59]. We considered that Rand-GHP is a valid measure for investigating the MID as a result of an intervention.

### Strength and limitations of the study

The strength of our study is that we contributed to the scientific literature on PROMs and explored the use of MIDs in the field of severe mental illness. We need to be cautious about drawing definite conclusions based on our findings because of the relatively low sample size and the significant gender confounder. The statistical power of the results is low and our sample might not be good representation of the population of persons with severe mental illness. More men living in a supported housing facility might coincidently determine the variance in our observed scores. In a confirmative trial or in other existing datasets with a bigger sample, this study might necessarily be repeated. Although our sample size was small, it was large enough (> 40) to detect correlation coefficients of 0.50 or higher with a power of 96% [47] and it, therefore, properly based the MID-anchor calculations. Another strength of our study is that we were able to include non-completer participants with a low attendance rate, which makes the findings more realistic.

On one hand, our interviewer-administered method of data collection can be considered as a limitation. The face-to-face interviews might have caused response bias, in terms of acquiescence bias [60], and also social desirability, which is stronger in women compared to men [61]. This might have influenced the gender effect difference in our study. Respondents may deliberately answer questions inaccurately, either by underreporting or overreporting of normative or stigmatized issues such as sexual behaviour or eating patterns [62, 63]. In our data, we did not find a gender difference in the response to the item of satisfaction with their sexual life in the QoL measure (MANSA), which is an issue that could cause shame and be influenced by social desirable bias. Therefore, we could not conclude that social-desirability bias did lead to the gender difference found in our study and nor could we definitely rule out the presence of this bias. This bias, just as with acquiescence bias, could have occurred in the baseline as well as in the endpoint interviews. Therefore, we expect that the change we saw can be considered a real change. The length of the questionnaires could have caused cognitive fatigue and biased the results, because we did not change the order of the different questionnaires. In a confirmative trial, randomizing the order might prevent this bias.

On the other hand, the face-to-face interviews might have prevented non-response bias, by preventing attrition. We estimated that too many of our participants would not respond to self-administered questionnaires. Only participants with a higher level of functioning might have completed the questionnaires, which could have caused bias. We decided we could better use the advantages of face-to-face interviews [28] as mentioned before in the ‘Materials and method’ section.

### Conclusions

Taking into account the low sample size and the gender covariate, we conclude with certain prudence that the MHRM was capable of showing the most relevant and meaningful change in persons with severe mental illness as a result of the IMR programme.

### Implications for further research

Our research can be used as an example of how to estimate MIDs in the context of people with severe mental illness. More research with a larger sample needs to be done to gain a more solid grounding for the MIDs. This research needs to account for the gender covariate. In future research on the effectiveness of interventions for people with severe mental illness, a recovery measure such as the MHRM should be used.

### Implications for further practice

In the search for scientific information that can convince clinicians to change their treatment practices and convince policy-makers to change their treatment guidelines, our findings can be used, with certain prudence, in shared decision-making processes. When an outcome on recovery is desired, a person with severe mental illness can be assigned to the IMR programme. A recovery measure such as the MHRM is able to measure the effect and should be used uniformly.

## Abbreviations

BSI: Brief Symptom Inventory
e-IMR: E-health version of the Illness Management and Recovery programme
ES: Effect size
IMR: Illness Management and Recovery programme
IMRS: Illness Management and Recovery Scales
MANSA: Manchester Short Assessment of Quality of Life
MANSA-1: The first question of the MANSA
MHRM: Mental Health Recovery Measure
MID: Minimal important difference
MID-anchor: Minimal important difference based on the anchor
MID-SD_c_: Minimal important difference based on the standard deviation of the change scores
MID-SEM: Minimal important difference based on the standard error of measurement
PAM: Patient Activation Measure
PROM: Patient-reported outcomes measure
QoL: Quality of life
Rand-GHP: Rand General Health Perception
Rand-HC: Rand Health Change
*r*_xx_: Test–retest coefficient index
SD: Standard deviation
SD_c_: Standard deviation of the change scores
SEM: Standard error of measurement

## References

[1] Slade M (2009). Personal recovery and mental illness, a guide for mental health professionals.

[2] Anthony WA (1993). Recovery from mental illness: The guiding vision of the mental health service system in the 1990s. Psychosocial Rehabilitation Journal.

[3] Barlow J, Wright C, Sheasby J, Turner A, Hainsworth J (2002). Self-management approaches for people with chronic conditions: a review. Patient Education and Counseling.

[4] Slooff, C., & van Alphen, G. (2012). Over de contraproductieve houding van de psychiatrie en de GGZ bij stigmatisering. In J. Dröes & C. Witsenburg (Eds.), Herstelondersteunende Zorg [Recovery Oriented Care]. Amsterdam: Uitgeverij SWP.

[5] Farkas M, Anthony WA (2010). Psychiatric rehabilitation interventions: A review. International Review of Psychiatry.

[6] Gingerich S, Mueser KT (2011). Illness Management & Recovery, Personalized Skills and Strategies for Those with Mental Illness, Implementation Guide.

[7] McGuire AB, Kukla M, Green A, Gilbride D, Mueser KT, Salyers MP (2014). Illness management and recovery: A review of the literature. Psychiatric Services (Washington, D.C.).

[8] Mueser KT, Meyer PS, Penn DL, Clancy R, Clancy DM, Salyers MP (2006). The Illness Management and Recovery program: Rationale, development, and preliminary findings. Schizophrenia Bulletin.

[9] Salyers MP, Rollins AL, McGuire AB, Gearhart T (2009). Barriers and facilitators in implementing illness management and recovery for consumers with severe mental illness: Trainee perspectives. Administration and Policy in Mental Health.

[10] Salyers MP, Godfrey JL, McGuire AB, Gearhart T, Rollins AL, Boyle C (2009). Implementing the Illness Management and Recovery program for consumers with severe mental illness. Psychiatric Services.

[11] Fujita E, Kato D, Kuno E, Suzuki Y, Uchiyama S, Watanabe A (2010). Implementing the illness management and recovery program in Japan. Psychiatric Services (Washington, D.C.).

[12] Färdig R, Lewander T, Melin L, Folke F, Fredriksson A (2011). A randomized controlled trial of the illness management and recovery program for persons with schizophrenia. Psychiatric Services (Washington, D.C.).

[13] Hasson-Ohayon I, Roe D, Kravetz S (2007). A randomized controlled trial of the effectiveness of the illness management and recovery program. Psychiatric Services (Washington, D.C.).

[14] Levitt AJ, Mueser KT, Degenova J, Lorenzo J, Bradford-Watt D, Barbosa A (2009). Randomized controlled trial of illness management and recovery in multiple-unit supportive housing. Psychiatric services (Washington, D.C.).

[15] Jayadevappa R, Cook R, Chhatre S (2017). Minimal important difference to infer changes in health-related quality of life—A systematic review. Journal of Clinical Epidemiology.

[16] Guyatt GH, Walter S, Norhfm G (1987). Measuring change over time: assessing the usefulness of evaluative instruments. Journal of Chronical Disease.

[17] Revicki DA, Hays RD, Cella D, Sloan J (2008). Recommended methods for determining responsiveness and minimally important differences for patient-reported outcomes. Journal of Clinical Epidemiology.

[18] Angst F, Aeschlimann A, Angst J (2017). The minimal clinically important difference raised the significance of outcome effects above the statistical level, with methodological implications for future studies. Journal of Clinical Epidemiology.

[19] Guyatt GH, Osoba D, Wu AW, Wyrwich KW, Norman GR, Aaronson N (2002). Methods to explain the clinical significance of health status measures. Mayo Clinic Proceedings.

[20] King MT (2011). A point of minimal important difference (MID): A critique of terminology and methods. Expert Review of Pharmacoeconomics and Outcomes Research.

[21] Den Oudsten BL, Zijlstra WP, De Vries J (2013). The minimal clinical important difference in the World Health Organization Quality of Life instrument—100. Supportive Care in Cancer.

[22] Turner D, Schünemann HJ, Griffith LE, Beaton DE, Griffiths AM, Critch JN, Guyatt GH (2010). The minimal detectable change cannot reliably replace the minimal important difference. Journal of Clinical Epidemiology.

[23] Beentjes TAA, Goossens PJJ, Vermeulen H, Teerenstra S, Nijhuis-van der Sanden MWG, van Gaal BGI (2018). E-IMR: e-health added to face-to-face delivery of Illness Management and Recovery programme for people with severe mental illness, an exploratory clustered randomized controlled trial. BMC Health Services Research.

[24] Beentjes TAA, van Gaal BGI, Goossens PJJ, Schoonhoven L (2016). Development of an e-supported illness management and recovery programme for consumers with severe mental illness using intervention mapping, and design of an early cluster randomized controlled trial. BMC Health Services Research.

[25] Delespaul PH, Dutch consensus group SMI (2013). Consensus regarding the definition of persons with severe mental illness and the number of such persons in the Netherlands. Tijdschrift voor Psychiatrie.

[26] Rotondi AJ, Eack SM, Hanusa BH, Spring MB, Haas GL (2015). Critical design elements of E-health applications for users with severe mental illness: Singular focus, simple architecture, prominent contents, explicit navigation, and inclusive hyperlinks. Schizophrenia Bulletin.

[27] MacKin RS, Delucchi KL, Bennett RW, Areán PA (2011). The effect of cognitive impairment on mental healthcare costs for individuals with severe psychiatric illness. American Journal of Geriatric Psychiatry.

[28] Streiner DL, Norman GR, Cairney J (2008). Health Measurement Scales, a practical guide to their development and use.

[29] Mueser, K. T., Gingerich, S., Salyers, M. P., McGuire, A. B., Reyes, R. U., & Cunningham, H. (2005). Illness Management and Recovery (IMR) Scales. In T. Campbell-Orde, J. Chamberlin, J. Carpenter, & H. S. Leff (Eds.), Measuring the promise: A compendium of recovery measures Volume II (pp. 32–35 & 124–132). Cambridge, MA: Human Services Research Institute.

[30] Färdig R, Lewander T, Fredriksson A, Melin L (2011). Evaluation of the Illness Management and Recovery Scale in schizophrenia and schizoaffective disorder. Schizophrenia Research.

[31] Goossens PJJ, Beentjes TAA, Knol S, Salyers MP, de Vries SJ (2017). Investigating the reliability and validity of the Dutch versions of the illness management and recovery scales among clients with mental disorders. Journal of Mental Health (Abingdon, England).

[32] Hasson-Ohayon I, Roe D, Kravetz S (2008). The psychometric properties of the Illness Management and Recovery scale: Client and clinician versions. Psychiatry Research.

[33] Salyers MP, Godfrey JL, Mueser KT, Labriola S (2007). Measuring illness management outcomes: A psychometric study of clinician and consumer rating scales for illness self management and recovery. Community Mental Health Journal.

[34] Hibbard JH, Mahoney ER, Stockard J, Tusler M (2005). Development and testing of a short form of the patient activation measure. Health Services Research.

[35] Moljord IEO, Lara-Cabrera ML, Perestelo-Pérez L, Rivero-Santana A, Eriksen L, Linaker OM (2015). Psychometric properties of the Patient Activation Measure-13 among out-patients waiting for mental health treatment: A validation study in Norway. Patient Education and Counseling.

[36] van Nieuwenhuizen C, Wilrycx G, Moradi M, Brouwers E (2014). Psychometric evaluation of the Dutch version of the mental health recovery measure (MHRM). The International Journal of Social Psychiatry.

[37] Bullock, W. (2009). The Mental Health Recovery Measure (MHRM): Updated Normative Data and Psychometric Properties Overview. Updated Normative Data and Psychometric Properties. Toledo, OH. Retrieved from https://www.utoledo.edu/al/psychology/pdfs/MHRM_12-09.pdf

[38] Derogatis LR, Melisaratos N (1983). The Brief Symptom Inventory-an introductory report. Psychological Medicine.

[39] de Beurs E, Zitman F (2006). De Brief Symptom Inventory (BSI). De betrouwbaarheid en validiteit van een handzaam alternatief voor de SCL-90. [The Brief Symptom Inventory, reliability and validity of a conveniant alternative]. Maandblad Geestelijke volksgezondheid.

[40] Priebe S, Huxley P, Knight S, Evans S (1999). Application and Results of the Manchester Short Assessment of Quality of Life (MANSA). International Journal of Social Psychiatry.

[41] Birgisson, Ó. G., & Sigurðsson, B. H. (2016). Psychometric Properties of the Icelandic Manchester Short Assessment of Quality of Life (MANSA) and its Possible Utility in Iceland. Vísindadagur meistaranema í klínískri sálfræði við Háskólann í, (May). https://doi.org/10.13140/RG.2.2.22634.31681

[42] Hays, R. D., Sherbourne, C. D., & Mazel, R. M. (1993). The RAND 36-Item Health Survey 1.0. *Health Economics, 2*(3), 217–227. 10.1002/hec.4730020305 8275167

[43] van der Zee, K. I., Sanderman, R., Heyink, J. W., & de Haes, H. (1996). Psychometric Qualities of the RAND 36-Item Health Survey 1.0: A of Multidimensional Measure of General Health Status. *International Journal of Behavioral Medicine, 3*(2), 104–122. 10.1207/s15327558ijbm0302_2 16250758

[44] IBM, C.  (2015). IBM SPSS Statistics for Macintosh, Version 23.

[45] Revicki DA, Cella D, Hays RD, Sloan JA, Lenderking WR, Aaronson NK (2006). Responsiveness and minimal important differences for patient reported outcomes. Health and Quality of Life Outcomes.

[46] Field A (2013). Discovering Statistics Using IBM SPSS Statistics.

[47] Portney, L., & Watkins, M. (2015). *Foundation of Clinical Research, application to practice*. (3rd Edn.). Upper Sadle River, NJ: Pearson Education, Inc.

[48] Polit DF, Beck CT (2016). Nursing research: Generating and assessing evidence for nursing practice.

[49] Johnston BC, Ebrahim S, Carrasco-Labra A, Furukawa TA, Patrick DL, Crawford MW (2015). Minimally important difference estimates and methods: A protocol. British Medical Journal Open.

[50] Norman GR, Sloan JA, Wyrwich KW (2003). Interpretation of changes in health-related quality of life: The remarkable universality of half a standard deviation. Medical Care.

[51] Nahler, G. (2009). Standardized Response Mean (SRM). In Dictionary of Pharmaceutical Medicine (pp. 173–173). Vienna: Springer. https://doi.org/10.1007/978-3-211-89836-9_1323

[52] Wyrwich KW, Bullinger M, Aaronson N, Hays RD, Patrick DL, Symonds T (2005). Estimating clinically significant differences in quality of life outcomes. Quality of Life Research.

[53] McGuire AB, Luther L, White D, White LM, McGrew J, Salyers MP (2014). The “Critical” elements of illness management and recovery: Comparing methodological approaches. Administration and Policy in Mental Health.

[54] van Langen WJM, Beentjes TAA, van Gaal BGI, Nijhuis-van der Sanden MWG, Goossens PJJ (2016). How the illness management and recovery program enhanced recovery of persons With Schizophrenia and other psychotic disorders: A qualitative study. Archives of Psychiatric Nursing.

[55] Banaji, M. R., & Hardin, C. (1994). Affect and Memory in Retrospective Reports. In N. Schwarz & S. Sudman (Eds.), Autobiographical Memory and the Validity of Retrospective Reports (pp. 71–86). New York: Springer. https://doi.org/10.1007/978-1-4612-2624-6_6

[56] Coughlin SS (1990). Recall bias in epidemiologic studies. Journal of Clinical Epidemiology.

[57] Buunk AP, Gibbons FX (2007). Social comparison: The end of a theory and the emergence of a field. Organizational Behavior and Human Decision Processes.

[58] Bruin, M. De, Kok, G., & Schaalma, H. (2007). Coding manual for behavioral change techniques. Retrieved from http://www.biomedcentral.com/content/supplementary/1748-5908-7-92-S2.pdf

[59] van Houtum L, Rijken M, Heijmans M, Groenewegen P (2013). Self-management support needs of patients with chronic illness: Do needs for support differ according to the course of illness?. Patient Education and Counseling.

[60] Cabitza F, Dui LG, Banfi G (2019). PROs in the wild: Assessing the validity of patient reported outcomes in an electronic registry. Computer Methods and Programs in Biomedicine.

[61] Hebert JR, Ma Y, Clemow L, Ockene IS, Saperia G, Stanek EJ (1997). Gender differences in social desirability and social approval bias in dietary self-report. American Journal of Epidemiology.

[62] Catania JA, Gibson DR, Chitwood DD, Coates TJ (1990). Methodological problems in AIDS behavioral research: Influences on measurement error and participation bias in studies of sexual behavior. Psychological Bulletin.

[63] Kelly CA, Soler-Hampejsek E, Mensch BS, Hewett PC (2013). Social desirability bias in sexual behavior reporting: Evidence from an interview mode experiment in rural Malawi. International Perspectives on Sexual and Reproductive Health.

